# Supplementary material for: Tumor Microenvironment, HLA Class I and APM Expression in HPV-Negative Oral Squamous Cell Carcinoma
Source: Cancers (Basel). 2021 Feb 4;13(4):620. doi: 10.3390/cancers13040620 (PMC7914856; doi:10.3390/cancers13040620)
Supplement: Supplementary file 1 [file cancers-13-00620-s001.pdf]

cancers

MDPI

Supplementary materials

Tumor Microenvironment, HLA Class I and APM Expression in HPV-Negative Oral Squamous Cell Carcinoma

Claudia Wickenhauser [1,†], Daniel Bethmann [1,†], Matthias Kappler [2,†], Alexander Walter Eckert [2,3], André Steven [4], Jürgen Bukur [4], Bernard Aloysius Fox [5], Jana Beer [1] and Barbara Seliger [4,6,*]

[1] Institute of Pathology, University Hospital Halle (Saale), 06112 Halle (Saale), Germany; claudia.wickenhauser@uk-halle.de (C.W.); daniel.bethmann@uk-halle.de (D.B.); jana.beer@uk-halle.de (J.B.)
[2] Department of Oral, Maxillofacial and Plastic Surgery, University Hospital Halle (Saale), 06120 Halle (Saale), Germany; matthias.kappler@uk-halle.de (M.K.); alexander.eckert@uk-halle.de (A.W.E.)
[3] Department of Oral, Maxillofacial and Plastic Surgery, University Hospital of the Paracelsus Private Medical University of South Nuremberg, 90471 Nuremberg, Germany; alexander.eckert@klinikum-nuernberg.de (A.W.E.)
[4] Institute of Medical Immunology, University Hospital Halle (Saale), 06112 Halle (Saale), Germany; barbara.seliger@uk-halle.de (B.S.); andre.steven@uk-halle.de (A.S.); juergen.bukur@uk-halle.de (J.B.)
[5] Earle A. Chiles Research Institute, Robert W. Franz Cancer Research Center, Portland, OR 97213, USA; bernard.fox@providence.org (B.A.F.)
[6] Fraunhofer Institute for Cell Therapy and Immunology, 04103 Leipzig, Germany (B.S.)
* Correspondence: barbara.seliger@uk-halle.de; Tel.: +49-(0)-345-557-4054; Fax: +49-(0)-345-557-4055
† These authors contributed equally to this work.

Citation: Wickenhauser, C.; Bethmann, D.; Kappler, M.; Eckert, A.W.; Steven, A.; Bukur, J.; Fox, B.A.; Beer, J.; Seliger, B. Tumor Microenvironment, HLA class I and APM Expression in HPV-Negative Oral Squamous Cell Carcinoma. *Cancers* **2021**, *13*, 620. https://doi.org/10.3390/cancers13040620

Academic Editor: David Wong
Received: 8 January 2021
Accepted: 26 January 2021
Published: 4 February 2021

Publisher's Note: MDPI stays neutral with regard to jurisdictional claims in published maps and institutional affiliations.

Copyright: © 2021 by the authors. Licensee MDPI, Basel, Switzerland. This article is an open access article distributed under the terms and conditions of the Creative Commons Attribution (CC BY) license (http://creativecommons.org/licenses/by/4.0/).

Supplementary materials

Table S1. Concordant membranous HLA-I HC and β2-m expression with HLA-I surface expression in FFPE vs. frozen OSCC lesions.

cases	FFPE specimen		fresh frozen specimen
	**HC: IRS**	**β2-m: IRS**	**HLA-A/B/C: IRS**
high membranous HLA expression			
Case # 47	9	6	8
Case # 53	9	6	6
Case # 64	8	6	8
Case # 100	8	8	8
Case # 263	8	8	8
low / negative membranous HLA expression			
Case # 136	0	0	1
Case # 148	0	1	0
Case # 151	0	1	0
Case # 182	0	2	3
Case # 202	0	2	2

IRS: immune reactive score.Staining patterns of HLA-I HC and β2-m on FFPE specimen with known HLA expression status (concordant membranous positivity) were compared to HLA-I surface expression on fresh frozen tissues using the W6/32 antibody recognizing the trimeric HLA-I complex.

Table S2. Primers used for analyses of HLA-I and APM component expression.

primer	sequence	Tm
TAP1 FWD*	GGA ATC TCT GGC AAA GTC CA	60° C
TAP1 REV**	TGG GTG AAC TGC ATC TGG TA	60° C
TAP2 FWD	CCA AGA CGT CTC CTT TGC AT	60° C
TAP2 REV	TTC ATC CAG CAG CAC CTG TC	60° C
β2-m FWD	CTC GCG CTA CTC TCT CTT	60° C
β2-m REV	AAG ACC AGT CCT TGC TGA	60° C
HLA-A, -B, -C FWD	GCC TAC CAC GGC AAG GAT TAC	60° C
HLA-A, -B, -C REV	GGT GGC CTC ATG GTC AGA GA	60° C
LMP2 FWD	TGC TGC ATC CAC ATA ACC AT	60° C
LMP2 REV	TGT GCA CTC TCT GGT TCA GC	60° C
LMP10 FWD	GGG CTT CTC CTT CGA GAA CT	60° C
LMP10 REV	CAG CCC CAC AGC AGT AGA TT	60° C

*fwd = forward; **rev = reverse.

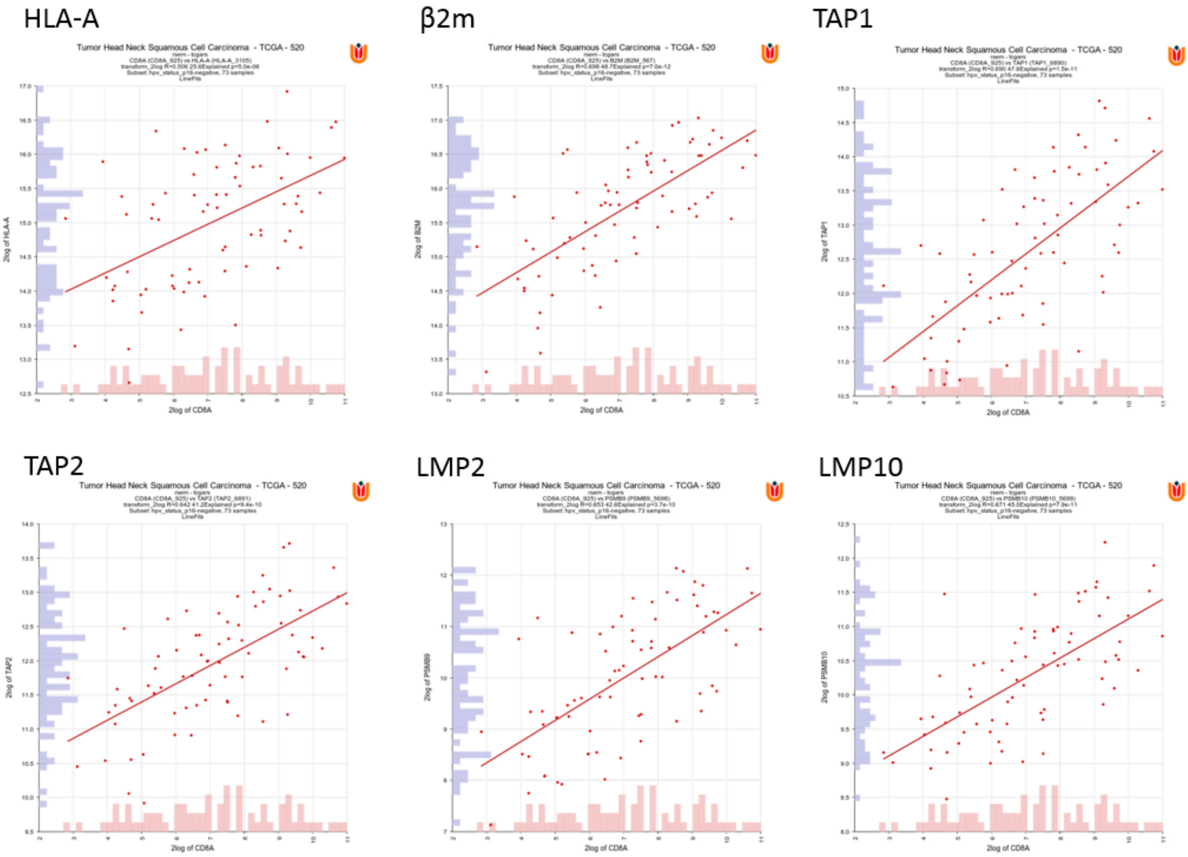

Figure S1. ***In silico*** **correlation of CD8 mRNA expression with HLA-A, β_2-m and APM components.** mRNA expression of CD8 was correlated with the mRNA expression of HLA-A, β_2-m, TAP1, TAP2, LMP2 and LMP10 in tumor material of HVP$^-$ HNSCC patients of the TCGA cohort (n=73). The histograms show the distribution of the patients as a xy-plot with the histogram and linear fit options. The p-value is given in the header of every histogram.

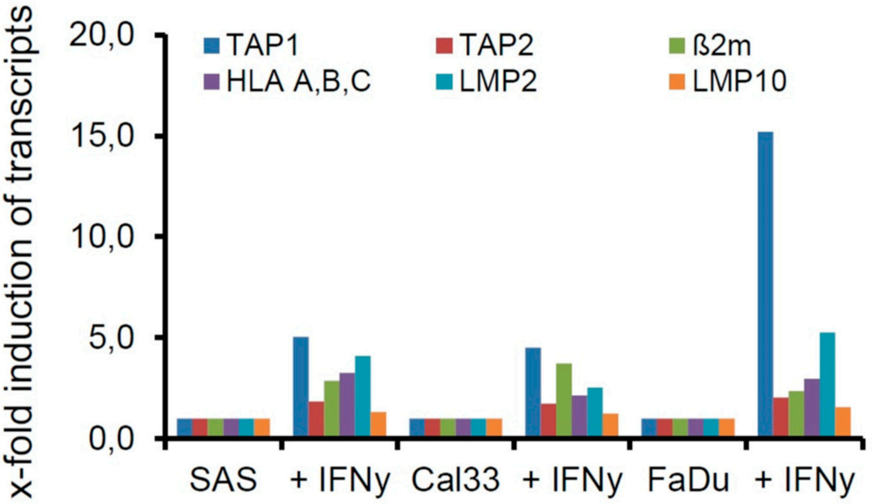

Ct value of APM component transcription

	SAS	SAS+IFNy	Cal33	Cal33+IFNy	FaDu	FaDu+IFNy
ß2m	8	9	9	9	8	9
HLA A,B,C	5	7	7	8	7	8
TAP1	6	7	7	8	6	8
TAP2	5	5	5	6	4	5
LMP2	7	8	8	9	7	9
LMP10	5	5	5	6	4	6

Ct value	10-12	12-14	14-16	16-18	18-20	20-22	22-24	24-26	>26
	9	8	7	6	5	4	3	2	1

(A)

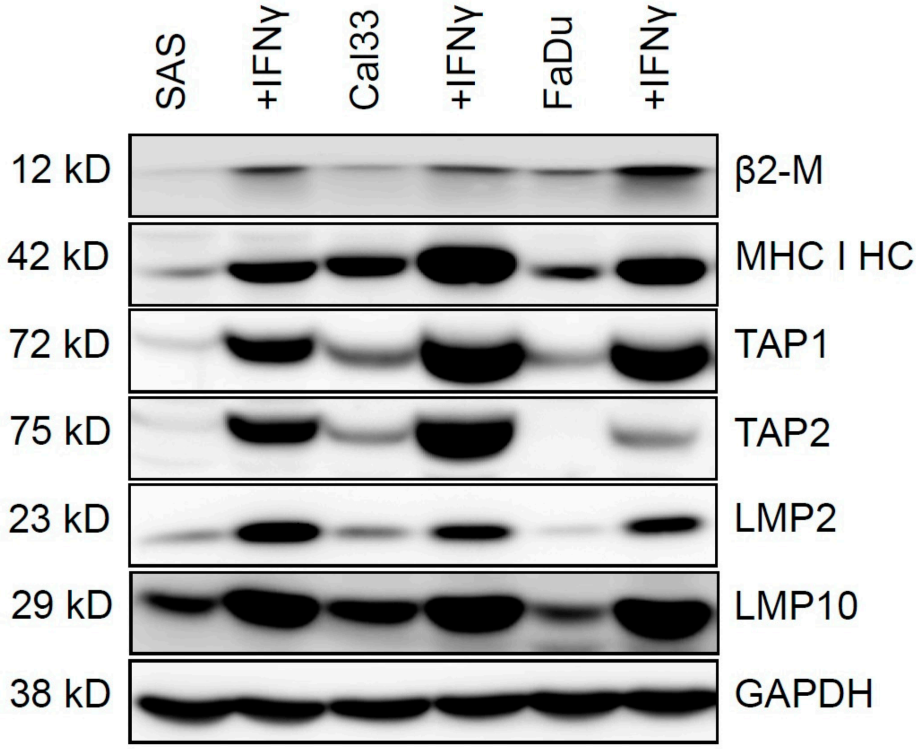

MHC class I surface expression as mean specific fluorescence intensity

Cell line	MFI (constitutive)	average deviation	MFI (IFN-γ)	average deviation
SAS	2.2	± 0.4	3.8	± 0.4
Cal33	3.5	± 0.8	6.9	± 1.7
FaDu	3.2	± 0.9	10.9	± 1.9

* mean of three independent replicates

(B)

Figure S2. Constitutive and IFN-γ mediated upregulation of HLA-I APM component expression in HNSCC cell lines.A Effect of IFN- treatment on mRNA expression of HNSCC cell lines.A: HNSCC cell lines were left untreated or treated for 48 h with IFN-γ (200 U/ml) as described in Materials and Methods before qPCR was performed. The data represent the relative mRNA expression levels of the different APM components normalized to that of the housekeeping gene ALAS1. The transcription levels of untreated HNSCC cell lines were set to one, and respective expression levels of IFN-γ-treated cells were calculated. Ct values of HLA-/APM component transcription and their classification are given. B Effect of IFN- on protein expression of HNSCC cell lines.B: Western blot analysis using 50 µg protein lysate/lane of three different HNSCC cell lines

(SAS, Cal33, FaDu) left untreated or treated for 48 h with IFN-γ (200 U/ml) was performed as described in Materials and Methods. The detection of GAPDH served as loading control. HLA-I surface expression of untreated and IFN-γ-treated HNSCC cell lines was performed as described and data expressed as MFI.
